# Modulation of TNFR 1-Triggered Inflammation and Apoptosis Signals by Jacaranone in Cancer Cells

**DOI:** 10.3390/ijms252413670

**Published:** 2024-12-20

**Authors:** Jie Liu, Yang Xu, Guobin Xie, Bingjie Geng, Renjing Yang, Wenjing Tian, Haifeng Chen, Guanghui Wang

**Affiliations:** School of Pharmaceutical Sciences, Xiamen University, Xiamen 361102, China; jieliu@xmu.edu.cn (J.L.); xu_yang@xmu.edu.cn (Y.X.); xieguobin@xmu.edu.cn (G.X.); 32320221154439@stu.xmu.edu.cn (B.G.); 32320230157401@stu.xmu.edu.cn (R.Y.); tianwj@xmu.edu.cn (W.T.)

**Keywords:** jacaranone, TNFR1, cIAP2, NF-κB, apoptosis

## Abstract

Jacaranone derived from *Senecio scandens*, a traditional Chinese medicine used for centuries, has been documented to exhibit anti-inflammatory and antiproliferative properties in various tumor cell lines. However, the mechanism of action and relationship between inflammation and apoptosis induced by jacaranone remain inadequately elucidated. In this study, the targets of jacaranone and cancer were identified from various databases, while potential targets and pathways were predicted through the analysis of the protein–protein interactions (PPI) network and pathway enrichment. Through a comprehensive network pharmacology analysis and corroborating experimental findings, we revealed that jacaranone induces tumor cell death by fine-tuning the tumor necrosis factor receptor 1 (TNFR1) downstream signaling pathway. TNFR1 serves as a key node that assembles into complexes I and II, regulating pathways including the nuclear factor (NF)-κB signaling pathway and the cell apoptosis pathway, which play crucial roles in cellular life activities. Jacaranone successfully guides survival signaling pathways to apoptotic mechanisms by inhibiting the assembly of complex I and promoting the formation of complex II. In particular, the main action mechanism of jacaranone lies in inducing the degradation of the inhibitor of apoptosis protein (cIAP)-2. cIAP-2 serves as an E3 ubiquitin ligase that ubiquitinates receptor-interacting serine/threonine-protein kinase 1 (RIPK1), thereby hindering the formation of complex I and effectively reducing the phosphorylation of Inhibitor of κB kinase (IKK) β. When the deubiquitylation process of RIPK1 is triggered, it may promote the formation of complex II, which ultimately leads to cell apoptosis. This fully demonstrates the key role of jacaranone in regulating TNFR1 complexes, especially through the degradation of cIAP-2. Taken together, jacaranone hinders the assembly of TNFR1 complex I and promotes the formation of complex II to induce apoptosis of cancer cells. Our findings unveil a novel mechanism underlying jacaranone, while also presenting a fresh approach for the development of new pharmaceuticals.

## 1. Introduction

According to data provided by the World Health Organization (WHO), cancer, including lung, colon, and breast cancer, continues to be a significant cause of mortality in numerous countries and is exhibiting an upward trend. Despite the current availability of several treatment options for cancer patients, ongoing research is being conducted on novel therapeutic agents. Extensive research has been conducted to identify pivotal targets or signaling pathways that hold potential for therapeutic interventions aimed at reducing fatality rates associated with these diseases. The utilization of herbal natural products has long served as a prolific reservoir of key compounds, playing an indispensable role in the realm of pharmaceutical exploration for centuries. Jacaranone, which is the methyl ester derivative of quinolacetic acid, was initially isolated from *Jacaranda caucana* (Bignoniaceae) [1]. Consequently, a plethora of jacaranone derivatives have been isolated from diverse botanical sources, predominantly affiliated with the *Senecio* species [2,3,4]. The *Senecio* species exhibit primarily antibacterial, antioxidative, antiviral, and antineoplastic properties among others. These species have been traditionally employed in folk medicine for the treatment of inflammatory conditions, bacterial infections, arthritis, and rheumatic diseases [2,5]. Extensive investigations have been conducted on the diverse biological activities of jacaranone and its derivatives, encompassing cytotoxicity, antimicrobial effects, anti-inflammatory properties, antioxidant potential, sedative activity, antiprotozoal action [6,7,8,9], and the remarkable cytotoxic activity to against various cancer cell lines [10,11,12]. However, its mechanism of antitumor activity remains unexplored.

The ancient biological processes of inflammation and cellular death play a crucial role in determining the fate of cells. The tumor necrosis factor (TNF) is pivotal in regulating both inflammation and programmed cell death, facilitated by distinct signaling complexes [13]. The formation of complex I, which is enclosed by a membrane, occurs rapidly upon detection of TNFα and primarily leads to the activation of genes associated with inflammation. The assembly process of complex I initiates with the interaction between RIPK1 and the TNFR1-associated death domain protein (TRADD), both binding to the cytosolic region of the receptor TNFR1 [14]. This interaction then facilitates the recruitment of TNF receptor associated factor 2 (TRAF2) and cIAP1 as well as cIAP2, which are ubiquitin ligases. The determination of cell fate is influenced by the levels of cIAP1 and cIAP2 [15,16]. The cIAP1/2 E3 ligase complex K63 ubiquitylates RIPK1 in complex I [17], which then serves as a scaffold for the assembly of IKKα/IKKβ/IKKγ protein complexes to facilitate inhibitor of NF-κB (IκB) phosphorylation and activate canonical NF-κB signaling [18,19].

The primary reaction of cells to TNFα is not apoptosis. Cell death checkpoints, referred to as protective mechanisms, actively inhibit the cytotoxic effects of TNFα to protect the organism from its potential detrimental outcome. The first cell death checkpoint identified consists of pro-survival proteins that are upregulated in an NF-κB-dependent manner. This “NF-κB checkpoint” mainly functions by restricting the activity of caspase-8. Defects in the upstream components of the TNF signaling pathway necessary for NF-κB signaling (such as cIAP1/2) can disrupt the cell death checkpoint, thereby triggering apoptosis [20,21]. These proteins also play a crucial role in inhibiting caspase-8 activation within complex II. Complex II arises from the interaction between fas-associated death domain-containing protein (FADD), the components TRADD, and/or RIPK1, which dissociate from the receptor-bound complex I. Complex II serves as a platform within the cytoplasm for the activation of caspase-8, facilitating its binding and subsequent processing of downstream effector caspases that ultimately trigger apoptosis [22]. Relevance of the ubiquitin network linked to complex I is crucial for the activation of kinases regulating cell death checkpoints. Hence, any alterations in the ubiquitylation process of complex I, like hindering cIAP1 or cIAP2 activity, disrupt these cellular mechanisms and trigger TNF cytotoxicity. When both cIAP1 and cIAP2 were absent, RIPK1 polyubiquitination was impaired, resulting in decreased phosphorylation of IKKβ, and activation of caspase-8 [17,23,24]. The overexpression of cIAP1 and cIAP2 has been commonly detected in different types of cancer, indicating the significant possibility of utilizing IAP antagonism for precise cancer treatment [25,26].

In this study, the key targets and pathways of jacaranone for treating cancer were predicted through PPI network analysis and pathway enrichment analysis. Our findings revealed that jacaranone effectively suppressed the expression of an NF-κB reporter gene induced by TNFα in a concentration-dependent manner. Additionally, jacaranone hindered the nuclear translocation of p65 and degradation of IκBα triggered by TNFα. These inhibitory effects were also observed in vivo. The effects of jacaranone involve the induction of cIAP2 degradation, leading to the disruption of the ‘NF-κB death checkpoint’ and disassembly of TNFR1 signaling complex I. Jacaranone not only hinders the formation of complex I, but also enhances the assembly of TNFR1 signaling complex II, thereby promoting TNFα-induced apoptosis in cancer cells. Our findings highlight the antitumor potential of jacaranone, an active ingredient from traditional Chinese medicine, which may be a novel therapeutic agent against human cancer.

## 2. Results

### 2.1. Biological Enrichment and Pharmacological Network Analysis of Jacaranone and Cancer

**Figure 1:**
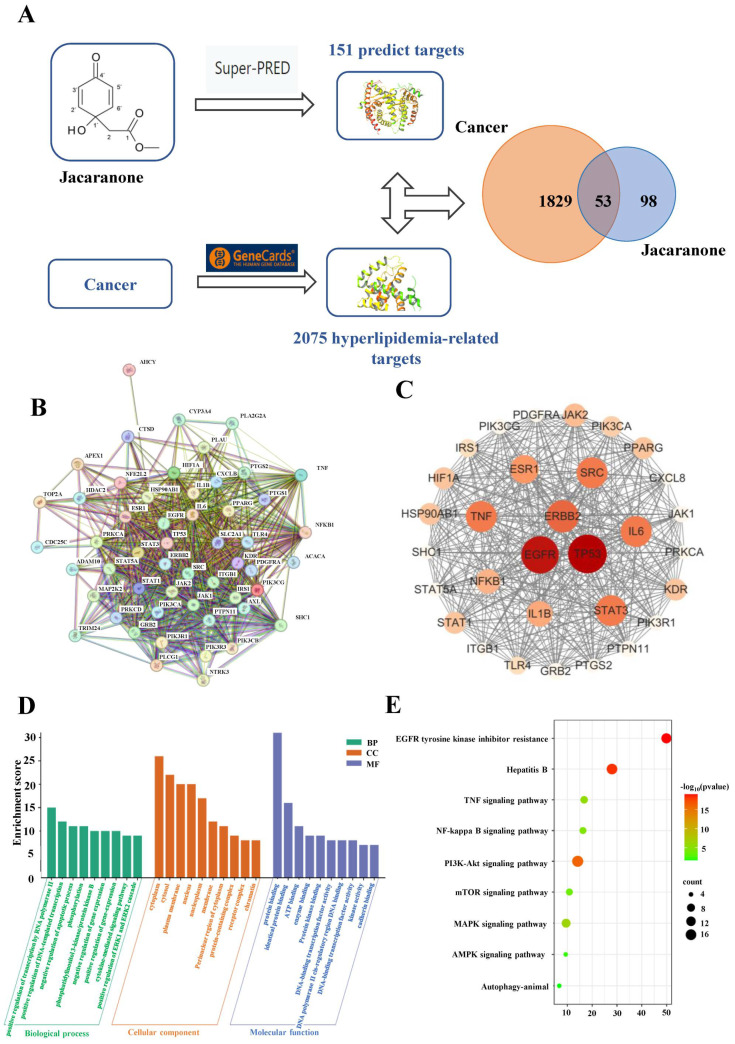
(**A**) Structure of jacaranone and the flowchart of this study is based on the methods of network pharmacology. (**B**) PPI network of common targets between jacaranone and cancer. (**C**) PPI network of core targets between jacaranone and cancer. The intensity of the dots reflects the degree of protein enrichment in C7-regulated pathways or functions. (**D**) Go function enrichment diagram of the common targets of jacaranone and cancer. (BP, Biological Process; CC, Cell Component; MF, Molecular Function) (**E**) KEGG pathway diagram of the common targets of jacaranone and cancer.

The bioactive compound jacaranone (Figure 1A), derived from *Senecio scandens* and with a long history of traditional use in Chinese medicine, exhibits potential inhibitory effects on cancer cells. However, the underlying mechanisms remain unknown. In this study, network pharmacology analysis was used to predict antitumor targets of jacaranone. The present study employed network pharmacology analysis to predict the potential antitumor targets of jacaranone. A total of 151 drug targets were identified using the SuperPred database. In addition, a comprehensive list of 2075 cancer-related genes were obtained from the GeneCards database, out of which 53 common targets (Figure 1A) were selected for further analysis. The jacaranone–cancer targets were inputted into the the search tool for the retrieval of interacting genes/proteins (STRING) database to obtain the PPI network (Figure 1B). The CentiScape software (version 3.10.1) was utilized to calculate the betweenness and closeness measures. Key proteins meeting the criteria of degree ≥ 26.49, closeness ≥ 0.013, and betweenness ≥ 25.19 were selected. A PPI network was constructed with 23 nodes and 409 edges using Cytoscape (version 2.2) (Figure 1C). The gene ontology (GO) enrichment analysis of the intersection targets revealed a total of 313 biological processes, 35 cellular components, and 76 molecular functions (Figure 1D). The Kyoto encyclopedia of genes and genomes (KEGG) enrichment analysis revealed a total of 140 KEGG pathways, including the NF-κB signaling pathway, the Phosphatidylinositol 3-kinase (PI3K)-Protein Kinase B (Akt) signaling pathway, the mechanistic target of rapamycin (mTOR) signaling pathway, the Mitogen-activated protein kinase (MAPK) signaling pathway, and the Adenosine 5‘-monophosphate (AMP)-activated protein kinase (AMPK) signaling pathway (Figure 1E).

### 2.2. Jacaranone Inhibits TNFα-Induced Activation of NF-κB

**Figure 2:**
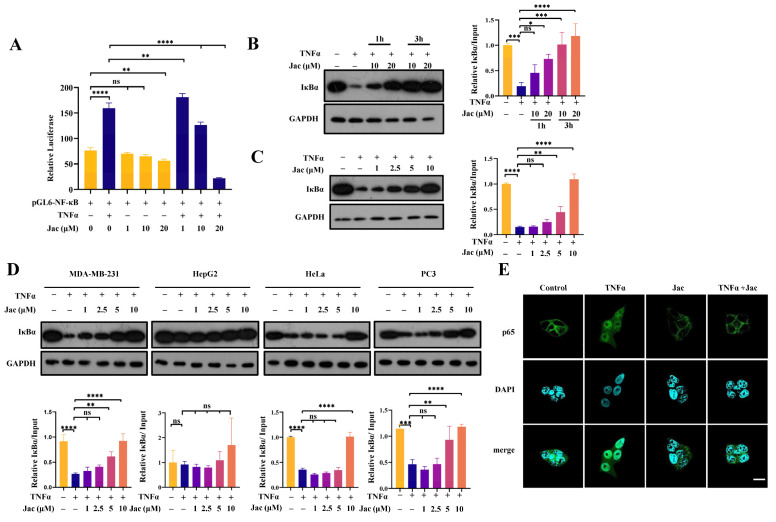
Jacaranone inhibits TNFα-induced activation of NF-κB. (**A**) The transcriptional activity of NF-κB induced by TNFα was inhibited by jacaranone. Plasmid pGL6-NF-κB and pRL-renilla were transfected into MCF7 cells, which were then treated with 20 ng/mL of TNFα along with varying concentrations (0, 1, 10 and 20 μM) of jacaranone for a duration of 24 h. Following transfection, luciferase activity was measured using a dual luciferin reporter gene kit, (n = 3). (**B**) Time-dependent inhibits the TNF-induced degradation of the IκBα effect of jacaranone. MCF7 cells treated with a vehicle or indicated concentration of jacaranone for 1 and 3 h. Subsequently, the cells were treated with TNFα at a concentration of 20 ng/mL for a period of 0.5 h. Western blotting techniques were used to detect the expression level of IκBα. In various experimental groups, Glyceraldehyde-3-Phosphate Dehydrogenase (GAPDH) and β-tubulin were utilized as internal controls. (**C**) Dose-dependent inhibits the TNF-induced degradation of the IκBα effect of jacaranone. MCF7 cells were pre-treated with various concentrations (0, 1, 2.5, 5 and 10 μM) of jacaranone for a duration of 3 h. Subsequently, the cells were treated with TNFα at a concentration of 20 ng/mL for a period of 0.5 h. The expression levels of IκBα were assessed using western blotting techniques. In various experimental groups, GAPDH and β-tubulin were utilized as internal controls. (**D**) Dose size inhibits the TNF-induced degradation of the IκBα effect of jacaranone in various cancer cells. MDA-MB-231, HepG2, HeLan and PC3 cells were pre-treated with various concentrations (0, 1, 2.5, 5 and 10 μM) of jacaranone for a duration of 3 h. Subsequently, the cells were treated with TNFα at a concentration of 20 ng/mL for a period of 0.5 h. The expression levels of IκBα were assessed using western blotting techniques. In various experimental groups, GAPDH and β-tubulin were utilized as internal controls. (**E**) The nuclear transport of p65 induced by TNFα is inhibited by jacaranone. MCF7 cells were pre-treated with 10 μM jacaranone for 3 h and then exposed to 20 ng/mL TNFα for 0.5 h before sample collection. Control and treated cells were subjected to staining with a p65 antibody, while the nucleus was stained with 4′,6-diamidino-2-phenylindole (DAPI), followed by observation under a confocal laser microscope, Scale bar, 10 μm. ns: no significance, * *p* < 0.05, ** *p* < 0.01, *** *p* < 0. 001, **** *p* < 0.0001. All blots above are representative of one of three experiments.

According to the network pharmacology analysis described above (Figure 1) and our cell-based high throughput screening for NF-κB signaling pathway inhibitors conducted using the Explorer^TM^ G3 workstation (Perkin Elmer, Waltham, MA, USA), it is indicated that jacaranone may exert its anticancer effects by targeting the NF-κB pathway. The impact of this compound on TNFα-induced NF-κB activation can be evaluated by conducting a test to quantify the activity of an NF-κB reporter gene in MCF7 cells (Figure 2A). The cells were transfected with a pGL6-NF-κB plasmid, followed by treatment with TNFα in the presence of varying concentrations of jacaranone. The results showed that jacaranone effectively inhibited the expression of the NF-κB reporter gene in a dose-dependent manner, confirming our expectations (Figure 2A). To further substantiate the inhibitory activity of jacaranone on the NF-κB pathway, we investigated it impacts on the expression of proteins associated with NF-κB in diverse cancer cell lines. The western blot analysis confirmed the anticipated time- and dose-dependent inhibitory effects of jacaranone on TNFα-induced IκBα degradation in breast cancer cell MCF7 cell (Figure 2B,C). Jacaranone also inhibits TNF-induced IκBα degradation in breast cancer cells (MDA-MB-231), cervical cancer cells (HeLa), and prostate cancer cells (PC3). However, its effect on liver cancer cells (HepG2) is notably weaker. Therefore, we primarily utilized breast cancer cells in our subsequent experiments (Figure 2D). The critical step in canonical NF-κB activation involves the translocation of the p65/p50 dimer from the cytoplasm to the nucleus, prompting us to perform an immunofluorescence assay. The results presented in Figure 2E demonstrate that jacaranone effectively inhibits the TNFα-induced translocation of p65 into the nucleus. The collective findings provide compelling evidence that jacaranone exerts a potent inhibitory effect on the NF-κB signaling pathway.

**Figure 3:**
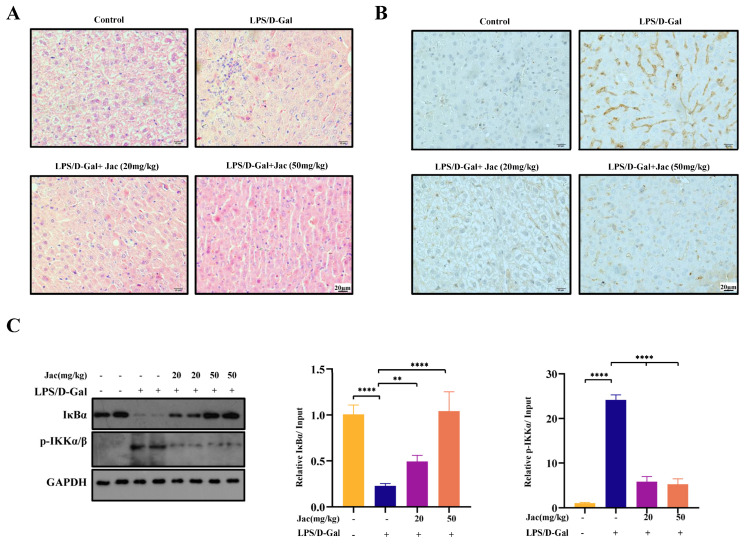
Jacaranone inhibits LPS/D-GalN induced inflammation in vivo. Mice were orally administered jacaranone at doses of 20 mg/kg or 50 mg/kg body weight, followed by an intraperitoneal injection of PBS or LPS/D-Gal (n = 4). (**A**,**B**) The liver sections were subjected to hematoxylin-eosin staining for the detection of necrosis and inflammatory cell infiltration (**A**), or CD68 staining for the identification of CD68 positive cells (**B**). Scale bar, 20 μm. (**C**) Western blot analysis was performed to assess the levels of IκBα and phosphorylated IKKα/β in control and treated tissues, with GAPDH and β-tubulin were served as a loading control for normalization purposes. ** *p* < 0.01, **** *p* < 0.0001. Blots above are representative of one of three experiments.

The findings prompted us to investigate the in vivo impact of jacaranone on the NF-κB signaling pathway. The administration of Lipopolysaccharide (LPS)/D-Galactosamine (GalN) triggers inflammatory responses associated with acute liver failure in mice. We expanded our research in LPS/GalN treated mice. In our study, mice were orally administered jacaranone at doses of 20 mg/kg or 50 mg/kg body weight, followed by an intraperitoneal injection of phosphate-buffered saline (PBS) or LPS (10 μg/kg) and D-GalN (500 mg/kg) separately. The Hematoxylin and Eosin (HE) staining revealed a significant infiltration of immune cells in the liver tissue of LPS/GalN-treated mice, which was effectively attenuated in a concentration-dependent manner following jacaranone intervention (Figure 3A). We investigated alterations in inflammatory cytokines and macrophages labeled with CD68. As depicted in Figure 3B, LPS/GalN induced activation of macrophages when compared to the control group, whereas treatment with jacaranone (20 and 50 mg/kg) exhibited a dose-dependent reduction in CD68-positive cells (Figure 3B). In addition, the administration of jacaranone (20 and 50 mg/kg) dose-dependently down regulated the degradation of IκBα and the phosphorylation of IKKα/β induced by LPS/GalN (Figure 3C). The collective findings of this study demonstrate that jacaranone effectively prevents endotoxic shock in mice and exerts inhibitory effects on the activation of the NF-κB signaling pathway in vivo.

### 2.3. Jacaranone Downregulates the Expression of cIAP2

**Figure 4:**
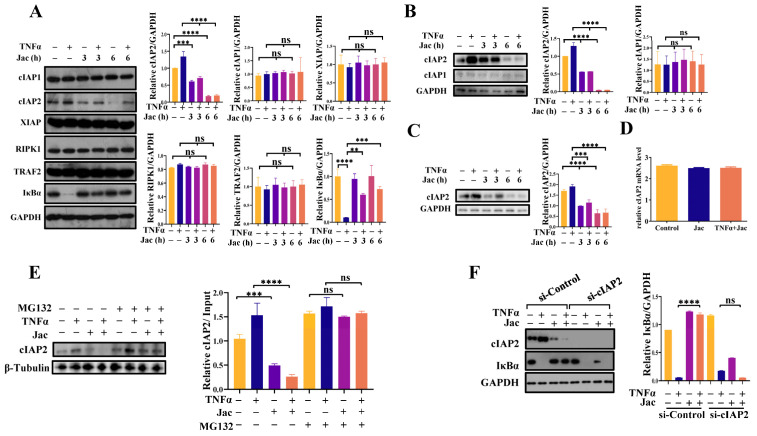
The degradation of cIAP2 induced by jacaranone played a crucial role in modulating inflammation and apoptosis. (**A**) The expression of cIAP2 protein was reduced by jacaranone treatment. MCF7 cells were treated with jacaranone (10 μM) for 3 h and 6 h, followed by TNFα (20 ng/mL) treatment for 0.5 h prior to sample collection. Western blot analysis was performed to assess the levels of cIAP1, cIAP2, XIAP, RIPK1, TRAF2, and IκBα. GAPDH was used as a loading control. (**B**,**C**) The expression of cIAP2 protein was reduced by jacaranone treatment. MDA-MB-231 (**B**) and HeLa (**C**) cells were treated with jacaranone (10 μM) for 3 h and 6 h, followed by TNF-α (20 ng/mL) treatment for 0.5 h prior to sample collection. Western blot analysis was performed to assess the levels of cIAP1 and cIAP2. GAPDH was used as a loading control. (**D**) The impact of jacaranone on cIAP2 mRNA levels was investigated in MCF7 cells. Cells were treated with jacaranone (10 μM) for 3 h, followed by TNFα (20 ng/mL) treatment for 0.5 h. RT-PCR was employed to assess the cellular mRNA levels. (**E**) The downregulation of cIAP2 expression induced by jacaranone can be effectively inhibited by MG132 treatment. MCF7 cells were pre-treated with 10 μM MG132 for 1 h prior to the administration of 10 μM jacaranone for 6 h. TNFα (20 ng/mL) was added 0.5 h before sample collection, and cIAP2 expression levels were assessed using immunoblotting techniques, with GAPDH and β-tubulin used as a reference for sample normalization. (**F**) The degradation of IκBα induced by TNFα is inhibited by jacaranone in a cIAP2-dependent manner. MCF7 cells were transfected with si-cIAP2. After 48 h of transfection, the cells were treated with jacaranone (10 μM) for 3 h and then stimulated with TNFα (20 ng/mL) for 0.5 h before sample collection. Western blotting was performed to analyze the levels of cIAP2 and IκBα, and GAPDH was used as a loading control. ns: no significance, ** *p* < 0.01, *** *p* < 0.001, **** *p* < 0.0001. All blots above are representative of one of three experiments.

The subsequent investigation aimed to determine if and how the TNFR1 signaling pathway is specifically regulated by jacaranone. Analysis of major TNFR1 signaling proteins in MCF7 cells revealed a significant downregulation in the expression of cIAP2 protein (Figure 4A). The degradation of cIAP2 was also induced by treatment with jacaranone in MDA-MB-231 cells (Figure 4B) and HeLa cells (Figure 4C). Further experiments demonstrated that jacaranone has no effect on mRNA of cIAP2 (Figure 4D). But reduction in expression could be prevented by the proteasome inhibitor MG132 (Figure 4E), suggesting that jacaranone may facilitate the proteasomal degradation of cIAP2. These findings suggest that decreased expression of cIAP2 is attributed to its degradation via the ubiquitin-proteasome pathway. To ascertain the significance of cIAP2 degradation in facilitating anti-inflammatory function of jacaranone, we employed a siRNA-mediated knockdown of cIAP2 expression and observed that jacaranone failed to suppress IκBα degradation in TNFα-induced cIAP2-deficient cells (Figure 4F), thereby underscoring the pivotal role played by cIAP2 in mediating jacaranone’s anti-inflammatory response.

### 2.4. Jacaranone Inhibits the TNF-Mediated NF-κB Signal Transduction Complex

**Figure 5:**
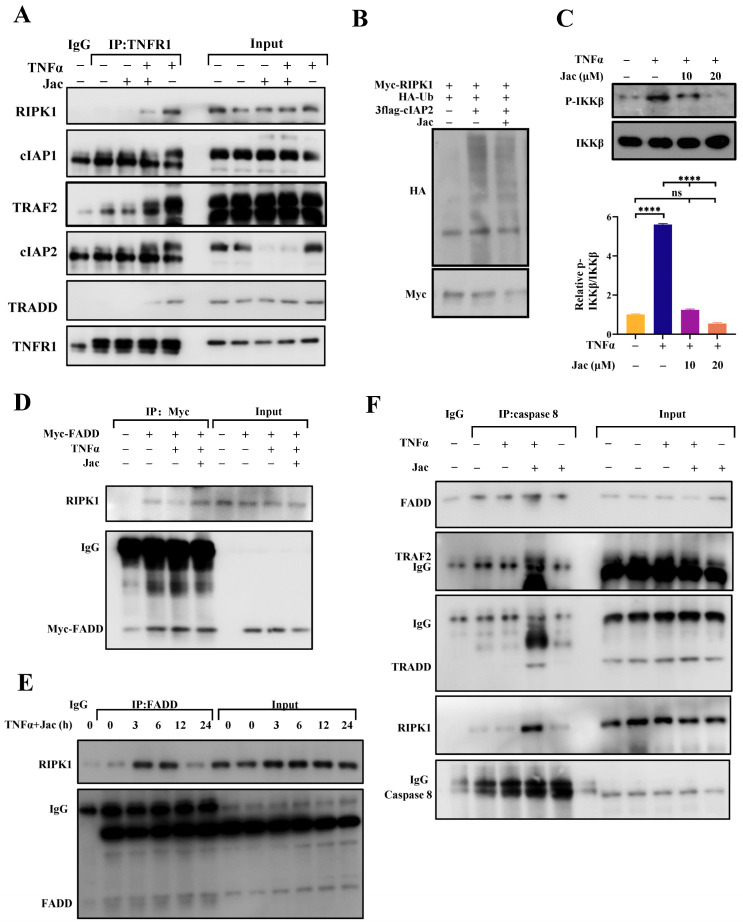
Jacaranone inhibits the formation of complex I and promotes the assembly of complex II. (**A**) The interaction among TNFR1 and RIPK1, TRAF2, cIAP1/2, and TRADD is inhibited by jacaranone. MCF7 cells were treated with 10 μM jacaranone for 3 h, followed by treatment with TNFα (20 ng/mL) for 0.5 h prior to sample collection. Immunoprecipitation was performed using a TNFR1 antibody, and the presence of RIPK1, TRAF2, cIAP1/2, TRADD, and TNFR1 proteins was detected through western blotting. (**B**) The ubiquitination of RIPK1 promoted by cIAP2 is inhibited by jacaranone. HEK293T cells were co-transfected with Myc-RIPK1, HA-Ub, and 3flag-cIAP2 plasmids, followed by treatment with jacaranone (10 μM) for 3 h after 24 h. (**C**) The phosphorylation of IKKβ is inhibited by jacaranone. MCF7 cells were pre-treated with 10 μM and 20 μM jacaranone for a duration of 3 h. Subsequently, the cells were treated with TNFα at a concentration of 20 ng/mL for a period of 0.5 h. The phosphorylation level of IKKβ were assessed using western blotting. Total IKKβ was used as an internal control. (**D**) 293T cells were transfected with Myc-FADD, treated with 10 μM jacaranone for 6 h, followed by treatment with TNFα (20 ng/mL) for 0.5 h prior to sample collection. Immunoprecipitation was performed using a Myc antibody, and the presence of RIPK1 and Myc were detected through western blotting. (**E**) MCF7 cells, treated with 10 μM jacaranone for 3, 6, 12, and 24 h, followed by treatment with TNFα (20 ng/mL) for 0.5 h. Immunoprecipitation was performed using a FADD antibody, and the presence of RIPK1 and FADD were detected through western blotting. (**F**) MCF7 cells, treated with 10 μM jacaranone for 6 h, followed by treatment with TNFα (20 ng/mL) for 0.5 h. Immunoprecipitation was performed using the caspase-8 antibody, and the presence of RIPK1, FADD, TRAF2, TRADD, and caspase-8 were detected through western blotting. ns: no significance, **** *p* < 0.0001. All blots above are representative of one of three experiments.

The significant anti-inflammatory role of cIAP2 in jacaranone prompted us to investigate the potential impact of jacaranone on the formation of TNFα-induced inflammatory complexes. Upon binding to TNFα, TNFR1 recruits the adaptor proteins TRADD, which subsequently recruits TRAF2 and RIPK1 K63-linked polyubiquitination. This polyubiquitination of RIPK1 constitutes a crucial step for IKKβ activation. The polyubiquitination of RIPK1 induced by TNFα is dependent on the interaction between cIAP1/2 and TRAF2. When the absence of both cIAP1 and cIAP2 was observed, RIPK1 polyubiquitination exhibited impairment, leading to a reduction in IKKβ phosphorylation and the subsequent activation of caspase-8 [17,23,24]. Co-immunoprecipitation experiments were conducted to assess the impact of jacaranone on the TNFα-induced interaction among components of the NF-κB signal transduction pathway. The interaction of TNFR1 with TRADD, TRAF2, RIPK1, and cIAP1/2 is induced by TNFα, as demonstrated in Figure 5A. However, jacaranone inhibits the assembly of complex I. The polyubiquitination of RIPK1 induced by TNFα is dependent on the cIAP1/2-TRAF2 interaction, thus we conducted an immunoprecipitation assay to examine the RIPK1 polyubiquitination. Overexpression of 3flag-cIAP2 leads to the induction of RIPK1 polyubiquitination, while jacaranone inhibits this effect (Figure 5B). The decrease in IKKβ activation was observed as a result of the reduction in RIPK1 polyubiquitin modification (Figure 5C). The results collectively demonstrate that jacaranone effectively impedes the formation of TNFR1 complex I and hinders the NF-κB signal transduction pathway activated by TNFα.

### 2.5. Jacaranone Promotes the Formation of Cytosolic Death Complexes

The NF-κB pathway serves as a crucial regulatory mechanism to suppress the cytotoxic effects of TNFα, thereby safeguarding the organism against potential deleterious consequences [20]. The inhibition of NF-κB can potentially induce cellular apoptosis, and this effect may be attributed to the formation of TNFR1 complex II. It is believed that the receptor dissociates TRADD and RIPK1, which subsequently recruit other proteins to form secondary death complexes. Our data demonstrate that jacaranone induces the release of RIPK1 and TRADD from TNFR1 (Figure 5A). Subsequently, we investigated whether jacaranone plays a role in the formation of TNFR1 complex II to initiate cell death. The induction of the RIPK1–FADD complex by jacaranone treatment in HEK-293T cells transfected with Myc-FADD was initially investigated (Figure 5D). Cell lysates were subjected to immunoprecipitation using an anti-Myc antibody and subsequently analyzed by immunoblotting with an anti-RIPK1 antibody. The formation of the RIPK1–FADD complex was promoted by jacaranone, as demonstrated in Figure 5D. This interaction was also assessed using an anti-FADD antibody in MCF7 cells (Figure 5E). Upon treatment with TNFα, MCF7 cells were exposed to 10 μM jacaranone for varying durations of 3, 6, 12, and 24 h. Experimental findings revealed a significant increase in the interaction between RIPK1 and FADD after 6 h of treatment with jacaranone, indicating its ability to facilitate the involvement of RIPK1 in complex II formation and subsequently activate the cell death receptor pathway. The caspase-8 antibody was additionally utilized to validate the formation of the death complex (Figure 5F). Jacaranone augments the interaction between caspase-8 and FADD, TRADD, RIPK1, and TRAF2, thereby demonstrating that jacaranone induces the assembly of complex II.

### 2.6. Jacaranone Potentiates the Apoptotic Effects Induced by TNFα

**Figure 6:**
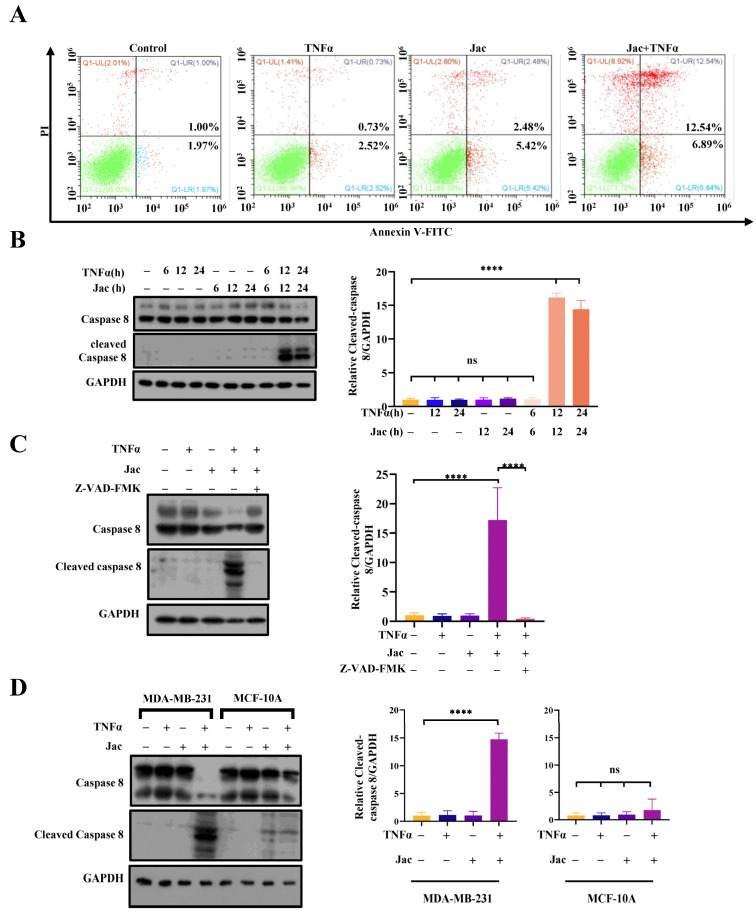
Jacaranone induces cancer apoptosis. (**A**) The apoptosis of jacaranone was evaluated using flow cytometry. MCF7 cells were exposed to a negative control, 20 ng/mL TNFα, 10 μM jacaranone, and a combination of both in a serum-free medium for a duration of 24 h. Subsequently, the cells were harvested and stained with propidium iodide and annexin V-FITC to detect apoptosis via flow cytometry, (n = 3). (**B**) The cleavage of caspase-8 induced by jacaranone was examined in MCF7 cells, both in the presence and absence of TNFα (20 ng/mL). Cells were treated with jacaranone (10 μM) for different time points: 6, 12, and 24 h for MCF7 cells. Western blot analysis was performed using equal amounts of total cell lysate to detect the expression levels of cleaved caspase-8 and PARP. GAPDH was used as a loading control. (**C**) The apoptosis of jacaranone can be suppressed by caspase inhibitors. HeLa cells were treated with TNFα (20 ng/mL), jacaranone (10 μM), and Z-VAD-FMK (10 μM) for 12 h, as depicted in the figure. Western blot analysis was conducted to assess the expression of cleaved caspase-8, while GAPDH was employed to determine sample size. (**D**) The cleavage of caspase-8 induced by jacaranone was examined in MDA-MB-231 and MCF-10A cells. Cells were treated with jacaranone (10 μM) for 24 h. Western blot analysis was performed using equal amounts of total cell lysate to detect the expression levels of cleaved caspase-8. GAPDH was used as a loading control. ns: no significance, **** *p* < 0.0001. All blots above are representative of one of three experiments.

The subsequent investigation focused on the potential of jacaranone to enhance TNFα-triggered apoptosis. Utilizing annexin V/propidium iodide (PI) dual staining (a widely used technique in the field of cell biology to assess apoptosis), it was observed that jacaranone significantly potentiated TNFα-induced apoptosis. The results demonstrated that exposure of MCF7 cells to the vehicle, TNFα, or jacaranone individually resulted in a relatively low level of apoptosis (2.97% for vehicle, 3.25% for TNFα, and 7.9% for jacaranone). However, simultaneous administration of both TNFα and jacaranone significantly increased apoptotic activity (19.43%) (Figure 6A). The crucial involvement of caspase-8, a renowned protease known for its pivotal role in facilitating apoptosis triggered by death receptors such as TNFR1, arises from its ability to promote both cell death processes. Our investigation aimed to ascertain the impact of jacaranone on the activation of caspase-8 induced by TNFα. The results demonstrated that treatment with either TNFα or jacaranone had minimal effects on caspase-8 activation. However, co-administration of TNFα and jacaranone resulted in a synergistic enhancement of caspase-8 activation, as indicated by the presence of cleaved caspase-8 (Figure 6B). Z-VAD-FMK is widely acknowledged as a potent inhibitor of caspases, effectively suppressing the activation of caspase-8 (Figure 6C). We also examined the apoptotic effect of jacaranone in MCF10A, a normal breast cell line. Our findings indicate that the combination of jacaranone and TNFα does not exhibit a synergistic enhancement in caspase-8 activation in MCF10A cells (Figure 6D). The results suggest that jacaranone selectively enhances the apoptotic effects induced by TNFα in cancer cells.

### 2.7. Jacaranone Exerts Antitumor Effect In Vivo

**Figure 7:**
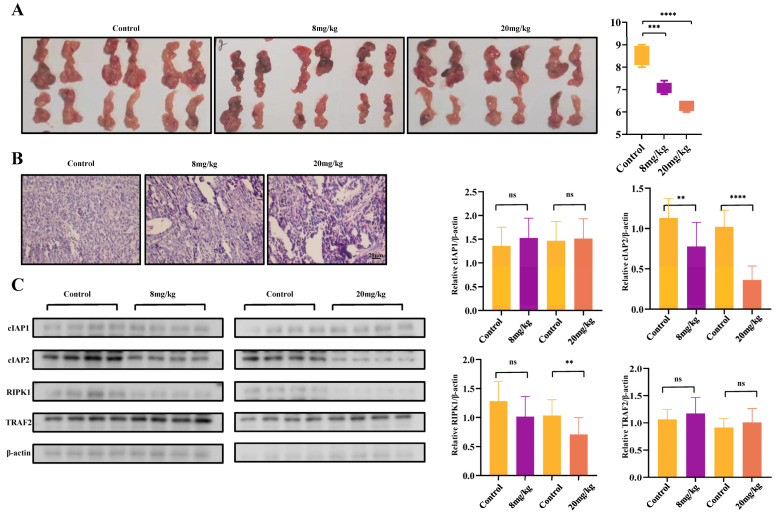
Jacaranone inhibits tumor growth in spontaneous breast cancer. The effect of jacaranone (8 mg/kg and 20 mg/kg by intraperitoneal injection) on MMTV-PyMT mice were evaluated, (n = 6). (**A**) The mammary tumor weight was assessed. Jacaranone-treated MMTV-PyMT mice developed smaller and lighter tumors than DMSO-treated mice. (**B**) Representative immune histological images from MMTV-PyMT mice treated with jacaranone or DMSO. Scale bar, 20 μm. (**C**) cIAP1/2, RIPK1, and TRAF2 expression were detected through western blotting in control and jacaranone-treated tumors tissues, while β-actin was used as a loading control. ns: no significance, ** *p* < 0.01, *** *p* < 0.001, **** *p* < 0.0001. All blots above are representative of one of three experiments.

The antitumor effects in vivo were evaluated in murine spontaneous breast-tumor model mouse mammary tumor virus promoter (MMTV)-Polyoma Virus middle T antigen (PyMT) and MCF7 xenografts in nude mice. In MMTV-PyMT mice, in comparison with the control group, there was a significant reduction in tumor weight at an intraperitoneal dosage of 8 mg/kg and 20 mg/kg (Figure 7A). HE staining results revealed that, in comparison with the control group, tumor tissues treated with an intraperitoneal dosage of 8 mg/kg and 20 mg/kg jacaranone exhibited cellular nucleus shrinkage and apoptosis (Figure 7B). Furthermore, the tumor tissues obtained from the control group and jacaranone’s groups were analyzed to determine the protein levels of the TNFR1 complex. The expression of cIAP2 was significantly reduced in the jacaranone-treated groups compared to the control group, as shown in Figure 7C, consistent with previous findings in cell lines. Additionally, no significant differences were observed for other proteins.

**Figure 8:**
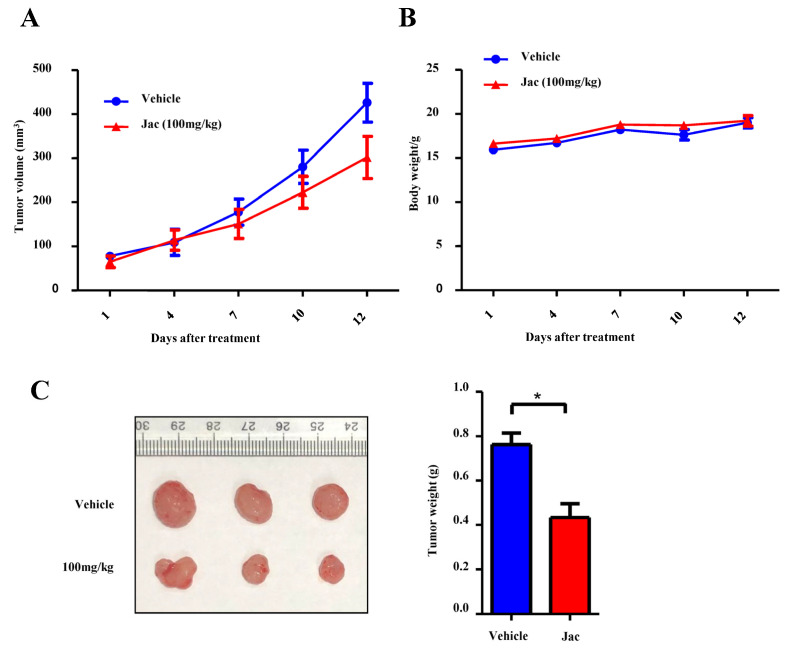
Jacaranone suppresses MCF-7 xenograft tumor growth in nude mice. MCF7 xenografted mice were intragastric administered with DMSO (1% in volume) or jacaranone (100 mg/kg) once every 2 days for a duration of 12 days, (n = 3). (**A**) Tumor volume was evaluated every 3 days, revealing that jacaranone-treated MCF7 xenografted mice exhibited smaller tumors compared to the DMSO-treated group. (**B**) Body weight was measured every 3 days, indicating no significant difference between jacaranone-treated and DMSO-treated MCF7 xenografted mice. (**C**) Following sacrifice, tumors were isolated and weighed, demonstrating that jacaranone reduced tumor weight significantly (* *p* < 0.05).

The treatment with jacaranone significantly attenuated tumor growth rates compared to the control group, as evidenced by the measurement of tumor volume in MCF7 xenografted mice (Figure 8A). The tumor weight of mice treated with 100 mg/kg jacaranone exhibited a significantly lower value compared to the control group (Figure 8C). The potential toxicity of jacaranone was evaluated by monitoring the body weight of mice every three days throughout the entire experiment. As depicted in Figure 8B, there were no significant alterations observed in the body weight of mice treated with jacaranone compared to the control group. Taken together, these findings suggest that jacaranone exhibits potent antitumor activity in an in vivo setting, with no significant toxicity observed even at doses as high as 100 mg/kg.

## 3. Discussion

The investigation of natural products has emerged as a pivotal avenue of research in recent years, aiming to identify potent antitumor constituents that exhibit optimal efficacy while minimizing toxicity [27]. The compound jacaranone is a naturally occurring benzoquinone molecule [28], which has been isolated from various plant species primarily belonging to the families of *Jacaranda Juss* and *Senecio.* The medicinal plant *Senecio scandens* Buch.-Ham is widely utilized in traditional Chinese medicine due to its pharmacological properties encompassing antibacterial, antiviral, trichomonas inhibition, and hepatoprotective effects. In recent years, an increasing number of studies have demonstrated the antitumor effects of jacaranone. Jacaranone exhibited the most potent antiproliferative activity against MDA-MB-231 (human breast cancer) and C33A (human cervical cancer) cells [10]. Jacaranone induces apoptosis in melanoma cells, demonstrating significant antitumor activity in vivo [11]. It has been also reported that jacaranone demonstrates inhibitory effects on the transcriptional activity of NF-κB [11]. Furthermore, this quinone has also been documented to possess antiproliferative properties against human tumor cell lines [10,11,12,28]. However, the mechanism of action and the relationship between inflammation and apoptosis induced by jacaranone remain unexplored. We have demonstrated for the first time that jacaranone promotes the formation of TNFR1 complex II and inhibits the activation of the NF-κB signaling pathway to suppress tumor growth. The cIAP2 plays a crucial role in facilitating the activation of TNF-induced NF-kB pro-survival signaling and inhibiting the TNFα-mediated extrinsic cell death pathway. cIAP2 is upregulated in multiple cancer cells, such as breast cancer [29] and colorectal cancer [30]. Degradation of cIAP2 may be a good target for tumor therapeutics. The expression of cIAP2 was reduced by suberoylanilide hydroxamic acid (SAHA) and epigallocatechin-3-gallate (EGCG), thereby attenuating the metastatic potential of TNBC through induction of the apoptotic pathway [29]. In this study, we demonstrate that jacaranone effectively induces the degradation of cIAP2 (Figure 4A–C). The cIAPs mediate the ubiquitylation of RIPK1 in complex I, functioning as a scaffold for assembling protein complexes consisting of IKKα/IKKβ/IKKγ, which are responsible for phosphorylating IκB and activating canonical NF-κB signaling. In the absence of both cIAP1 and cIAP2, RIPK1 exhibits deficient polyubiquitination, resulting in reduced phosphorylation of IKKβ and decreased survival rate of cancer cells. Accordingly, jacaranone inhibits cIAP2 overexpression induced RIPK1 ubiquitylation (Figure 5B) and phosphorylation of IKKβ (Figure 5C). Then, jacaranone effectively inhibits TNFα-induced NF-κB activation by suppressing IκBα degradation (Figure 2B–D), impeding NF-κB transcriptional activity (Figure 2A), and preventing p65 nucleation (Figure 2E) in different cancer cell lines. The results of our study demonstrated that jacaranone effectively inhibited the assembly of complex I induced by TNFα (Figure 5A).

TNFR1 signaling plays a multifaceted role in cancer development. The formation of TNFR1 complex I supports cell survival, whereas TNFR1 complex II induces apoptosis. However, the precise mechanisms underlying the transition between these TNFR1 complexes in cancer remain poorly elucidated. Histidine-rich glycoprotein (HRG) act in suppressing HCC via inclining TNFR1 to a pro-apoptotic cellular phenotype [31]. The discovery of drugs to modulate this switch has been proven insufficient. Based on both in vitro and in vivo experiments, jacaranone induces cancer cell apoptosis. On the one hand, jacaranone restricts the nuclear translocation of NF-κB in TNFα-treated cells, reducing cell proliferation mediated by TNFR1. On the other hand, jacaranone increases the formation of TNFR1 complex II, which directly leads to cell apoptosis. We show here that jacaranone enhances TNFα-induced apoptosis (Figure 6A) and induce caspase-8 activation (Figure 6B) in cancer cells. Furthermore, this phenomenon is susceptible to inhibition by caspase-8 inhibitors (Figure 6C). These findings strongly suggest a pivotal role for caspase-8 in the apoptotic process induced by jacaranone. Moreover, as an upstream regulator of the death receptor pathway, caspase-8 appears to be involved in the assembly of the death receptor complex (TNFR1 complex II). As well, the induction of cIAP2 degradation by jacaranone leads to the deubiquitylation of RIPK1 (Figure 5B), which plays a pivotal role in the assembly of the death-induced receptor complex and ultimately triggers apoptosis. Thereby leading us to postulate that jacaranone likely induces apoptosis through activation of the death receptor pathway. Our findings demonstrate that jacaranone effectively inhibits RIPK1 interaction with TNFR1 (Figure 5A) but enhances the interaction between RIPK1 and FADD (Figure 5D), providing compelling direct evidence for the pivotal role of jacaranone in Complex II formation. Moreover, jacaranone exerts regulatory control over the assembly of two distinct complexes via cIAP2, thereby modulating the delicate equilibrium between cellular survival and death, and ultimately influencing cell fate by inducing apoptosis.

The objective of this study was to assess the effectiveness and safety of jacaranone using xenotransplantation (Figure 8) and the murine spontaneous breast-tumor model MMTV-PyMT (Figure 7). The safety of jacaranone was also observed in normal cells (murine melanocytes and primary cultures of bone marrow macrophages from C57BL/6 mice) [11].The results demonstrate that jacaranone exhibits significant antitumor activity at non-toxic concentrations, indicating its potential as a safe and effective treatment option in preclinical models.

## 4. Materials and Methods

### 4.1. Jacaranone Preparation

The whole plant of *Senecio scandens* Buch.-Ham. ex D. Don. were collected from Quan Zhou city in Fujian Province, China. A voucher specimen (SES-201510) has been deposited in the School of Pharmaceutical Sciences, Xiamen University. The dried whole plant of *Senecio scandens* Buch.-Ham. ex D. Don. were crushed and extracted with 60% ethanol at three times (*w*/*v*) for 2 h. The solution was concentrated and subsequently separated by microporous resin column, silica gel column, ODS column, and Sephadex LH-20 column chromatography. The separated fraction was further purified by semi-preparative HPLC to yield jacaranone. The structure of jacaranone was identified by C13-NMR data comparison with the literature [12]. A large amount of jacaranone was purchased from Shanghai Mujin Biotechnology Co., Ltd. (Shanghai, China), with a purity of 95%.

### 4.2. Network Pharmacology

Jacaranone targets were predicted by SuperPred (https://prediction.charite.de/index.php, accessed on 23 July 2024) databases. The disease targets were identified using the search term “cancer” on GeneCards (https://www.genecards.org/, accessed on 23 July 2024). Drug–disease intersection targets were determined through a Venn diagram generated using the bioinformatics & evolutionary genomics website (https://www.bioinformatics.com.cn/, accessed on 23 July 2024). Among these, the promising therapeutic targets were submitted to STRING database (https://string-db.org/, accessed on 23 July 2024) and Cytoscape for PPI network formation. Using CentiScape (version 3.10.1) to calculate degree, betweenness and closeness, the targets were further screened based on these indicators. Thereafter, GO enrichment and KEGG pathway enrichment analyses were conducted by the David database. Finally, the image is drawn with the bioinformatics web (https://www.bioinformatics.com.cn/, accessed on 24 July 2024).

### 4.3. Cell Culture

The human breast cancer cell lines MCF7 and MDA-MB-231, the human hepatoma cell line HepG2, and the human embryonic kidney (HEK) 293T cells were cultured in Dulbecco’s Modified Eagle’s Medium (DMEM). The human prostate cancer cell line PC3 was routinely maintained in Roswell Park Memorial Institute (RPMI)-1640 medium. The human cervical cancer cell line HeLa was cultured in Minimum Essential Medium (MEM). All culture media were supplemented with 10% fetal bovine serum (FBS) (Gibco BRL, Rockville, MD, USA) and antibiotics (100 U/mL of penicillin and 100 mg/mL of streptomycin). All cell culture media and antibiotics were sourced from Shanghai BasalMedia Biotechnology Co., Ltd. (Shanghai, China).

### 4.4. Western Blot

The cells were lysed in RIPA buffer supplemented with a cocktail of phosphatase inhibitors and protease inhibitors. The proteins were resuspended in a sodium dodecyl sulfate (SDS) sample buffer containing 62 mM Tris-HCl (pH 6.8), 1 mM EDTA, 10% glycerol, 5% SDS, and 50 mM dithiothreitol. Subsequently, they were separated by SDS–PAGE. The protein was transferred from the gel onto the NC membrane. The membranes were blocked with a 5% non-fat skim milk solution in 1× TBS-T buffer for 1 h at room temperature (RT) and subsequently incubated overnight at 4 °C with primary antibodies (diluted to 1:1000). Following this, the membranes were exposed to horseradish peroxidase (HRP)-conjugated secondary antibodies (diluted to 1:10,000) for a duration of 1 h at RT. Protein levels were detected using the enhanced chemiluminescence (ECL) method. The western blot analysis revealed the expression levels of IKBα (cell signaling technology, #4812) (Danvers, MA, USA), p-IKKα/β (cell signaling technology, #2697) (Danvers, MA, USA), caspase-8 (cell signaling technology, #4790) (Danvers, MA, USA), cleaved-caspase-8 (cell signaling technology, #98134 and #9496) (Danvers, MA, USA), cIAP1 (cell signaling technology, #7065) (Danvers, MA, USA), cIAP2 (cell signaling technology, #3130) (Danvers, MA, USA), XIAP (cell signaling technology, #2042) (Danvers, MA, USA), RIPK1 (BD Biosciences, 551042) (Franklin Lakes, NJ, USA), TRAF2 (cell signaling technology, #4724), TNFR1 (Santa Cruz, sc-374185) (Santa Cruz, CA, USA), and FADD (cell signaling technology, #2782) (Danvers, MA, USA). The internal reference used in this study is GAPDH (cell signaling technology, #97166) (Danvers, MA, USA), β-actin (cell signaling technology, #4967) (Danvers, MA, USA), and β-Tubulin (cell signaling technology, #2146) (Danvers, MA, USA). The quantification was conducted using three distinct experimental samples.

### 4.5. Immunofluorescence

The cells were fixed onto coverslip glass using a 4% paraformaldehyde solution. Subsequently, the cells were permeabilized with 1% Triton X-100 in PBS at 37 °C for 15 min. Following this, the cells underwent incubation with a blocking solution (5% BSA in PBS) at RT for 1 h. The primary antibodies (diluted to a ratio of 1:100) were then applied to the cells overnight at 4 °C within the same blocking solution. Afterwards, TBS (composed of 50 mM Tris and 150 mM NaCl, pH level set at 7.4) was used to wash the cells. FITC-conjugated secondary antibodies (diluted to a ratio of 1:250) were subsequently applied to the cells at RT for 1 h. DAPI (diluted to a ratio of 1:2000) was used as an agent for counterstaining the cells. Finally, before imaging p65 using a Zeiss laser scanning confocal microscope (Oberkochen, Germany) it was necessary to use a mounting solution to securely seal the coverslip.

### 4.6. Flow Cytometry Assay

Following a 24 h treatment of MCF7 cells with jacaranone, the cells were resuspended in PBS and subjected to a 1 h incubation with PI and annexin V. Flow cytometry was employed to assess apoptosis.

### 4.7. RNA Extraction and Quantitative Real-Time Polymerase Chain Reaction (qRT-PCR)

The TRIzol reagent (Thermo Fisher Scientific, Waltham, MA, USA) was utilized for the extraction of total RNA and replicated three times. The RNA was subjected to reverse transcription using oligonucleotide primer (DT) 16 and M-MLV (Promega, Madison, WI, USA), resulting in the synthesis of first-strand cDNA. The qRT-PCR reaction was performed on the Agilent AriaMx system (Agilent Technologies, Santa Clara, CA, USA) utilizing PowerUp SYBR Green Master Mix (Thermo Fisher Science, Waltham, MA, USA). The amplification protocol consisted of a total of 40 cycles, with each cycle comprising a step at 95 °C for 10 s followed by another step at 60 °C for 30 s.

### 4.8. Co-Immunoprecipitation Analysis

The cells were washed with PBS and subsequently resuspended in 500 μL of NETN buffer, with a protein concentration of 1 µg/µL. A total of 10 μL lysis solution was used for input. The lysates were incubated with primary antibodies at a temperature of 4 °C for a period of 24 h. Anti-rabbit IgG antibodies were included as negative controls. Afterwards, the lysates were subjected to incubation with A/G agarose beads (obtained from Cell Signaling Technology in the Danvers, MA, USA) for a duration of 3 h followed by centrifugation. The beads were subsequently washed using NETN buffer, followed by the addition of 60 µL of 2× SDS loading buffer. For analysis, SDS–PAGE gels with a concentration of either 8% or 12%, 20 µL was utilized.

### 4.9. LPS/D-GalN-Induced Acute Liver Failure Model

A murine model of acute liver failure was established through intraperitoneal injection of LPS (10 μg/kg body weight) and D-GalN (500 mg/kg body weight). The mice were randomly divided into four groups (n = 4): a vehicle control group, an LPS/D-GalN group, and two jacaranone pretreatment groups with doses of 20 mg/kg and 50 mg/kg, respectively. In the jacaranone pretreatment groups, mice were orally administered jacaranone for 24 h prior to being injected with LPS and D-GalN separately. The vehicle control group received an equal volume of corn oil and sterile PBS. To assess liver injury, the mice were euthanized 8 h after the LPS/D-GalN injection, and liver tissue samples were collected. A small portion of the liver was fixed in a 4% paraformaldehyde solution, while the remaining tissue was frozen in liquid nitrogen and stored at −80 °C until analysis.

### 4.10. MMTV-PyMT Transgenic Mice and Jacaranone Treatments

The MMTV-PyMT mice, which exhibit spontaneous development of breast carcinoma, were randomly divided into four groups (n = 6): a control group (receiving an equal volume of solvent) and a jacaranone intraperitoneal injection group (8, 20 mg/kg). Tumor volume and body weight measurements were taken every 3 days for evaluation, along with an assessment of survival rates. After a therapy period lasting for 14 days, euthanasia was performed on the mice; their six internal organs and tumors were preserved in formalin for subsequent immunohistochemical staining as well as HE staining.

### 4.11. Xenotransplantation Experiment

The MCF7 cells were suspended in a cell suspension at a concentration of 1 × 10^7^/mL and transplanted into the right hindlimb of 5-week-old BALB/c nude mice (n = 3). Tumor development in the nude mice was monitored daily under strict aseptic conditions after tumor formation. The model was successfully established within 7 days when palpable irregular spots appeared were orally administered either DMSO or jacaranone (100 mg/kg) at a frequency of once every 2 days over a period of 12 days. Tumor measurements were taken every 3 days and tumor volumes were calculated using the formula: 0.52 × length × width^2^. After 12 days, the mice were euthanized, and the tumors were isolated to measure their weight.

### 4.12. Statistical Analysis

All the raw data were analyzed with GraphPad prism (version 9.0.0) software (GraphPad Software, La Jolla, CA, USA). Results are presented as mean values ± SEM. We first performed the Shapiro–Wilk test. If the normality test passed, we used an ordinary one-way ANOVA with Tukey post hoc. *p* < 0.05 was considered significant.

## 5. Conclusions

Overall, our findings demonstrate the effectiveness of jacaranone in attenuating NF-κB activity and inducing apoptosis in cancer cells. By inhibiting complex I and promoting the formation of complex II, jacaranone induces a shift from survival signaling to apoptotic machinery. Particularly of note is that jacaranone induces cIAP2 degradation. The reduction in cIAP2 expression caused by jacaranone leads to RIPK1 deubiquitylation, thereby inhibiting complex I formation and reducing IKKβ phosphorylation. As RIPK1-initiated cell death is also a crucial cellular response triggered by death factors, deubiquitylation of RIPK1 may induce complex II formation and ultimately result in cell apoptosis. Our study’s findings highlight the significant role played by jacaranone in regulating TNFR1 complexes through cIAP2 degradation.

## Data Availability

All data and models generated or used during the study appear in the submitted article.
